# Fluorescent Probes Detecting the Phagocytic Phase of Apoptosis: Enzyme-Substrate Complexes of Topoisomerase and DNA

**DOI:** 10.3390/molecules16064599

**Published:** 2011-06-03

**Authors:** Candace L. Minchew, Vladimir V. Didenko

**Affiliations:** 1 Baylor College of Medicine, Houston, TX 77030, USA; 2 Michael E. DeBakey Veterans Affairs Medical Center, 2002 Holcombe Boulevard, Research Building 109, Room 204, Houston, TX 77030, USA

**Keywords:** fluorescent protein-DNA probes, clearance of apoptotic cells, fluorescence labeling of phagocytosis, vaccinia topoisomerase I, phagolysosomes, DNase II-type breaks

## Abstract

In apoptosis, the initial self-driven suicide phase generates cellular corpses which are digested in the phagolysosomes of professional and amateur phagocytes during the subsequent waste-management phase. This ensures the complete elimination of the genetic material which often contains pathological, viral or cancerous DNA sequences. Although the phagocytic phase is critical for the efficient execution of apoptosis, there are currently few methods specifically adapted for its detailed visualization in the fixed tissue section format. To resolve this we developed new fluorescent probes for *in situ* research. The probes selectively visualize active phagocytic cells of any lineage (professional, amateur phagocytes or surrounding tissue cells) which engulf and digest apoptotic cell DNA. These fluorescent probes are the covalently-bound enzyme-DNA intermediates produced in a topoisomerase reaction with specific “starting” oligonucleotides. They detect a specific marker of DNase II cleavage activity, which occurs exclusively in phagolysosomes of the cells that engulfed apoptotic nuclei. The probes provide snap-shot images of the digestion process occurring in cellular organelles responsible for the actual execution of phagocytic degradation of apoptotic cell corpses. We applied the probes for visualization of the phagocytic reaction in tissue sections of normal thymus and in several human lymphomas. We also discuss the nature, stability and properties of DNase II-type breaks as a marker of phagocytic activity. This development provides a useful fluorescent tool for studies of pathologies where clearance of dying cells is essential, such as cancers, inflammation, infection and auto-immune disorders.

## 1. Introduction

Apoptosis is an orderly process of cellular elimination which ensures disassembly and disappearance of damaged cells. The complete removal of an apoptotic cell is a collective cellular event [1,2,3]. Starting as a self-initiated cellular suicide, it finishes as a cooperative clearance of apoptotic corpses by macrophages and other phagocytic cells. The full apoptotic cell clearance is divided into two phases: the self-driven cell disassembly and the externally-controlled elimination of apoptotic cell corpses by phagocytizing waste-management cells [1,4]. Efficient phagocytosis of apoptotic cells finalizes degradation of their DNA. This inhibits self-immunization, inflammation and the release of viral or tumor DNA [1,5]. The engulfment of dying cells by resident phagocytes is an important marker of apoptosis *in vivo* [6] and is an essential regulatory event in tissue development and homoeostasis [1].

Although detection of the phagocytic phase of apoptosis is essential for biomedicine, at the present time there are no specific *in situ* probes to accomplish this task. The currently used morphology-based microscopic assessments are time-consuming and imprecise. As a result, the detection and analysis of phagocytizing cells and discrimination between adherent and internalized apoptotic cells is difficult and labor-intense [7]. We set out to overcome this limitation. Our approach to detection of phagocytic clearance of apoptotic cells is based on labeling of a particular type of DNA breaks produced in this phase.

### 1.1. DNase II-Type Breaks and DNase I-Type Breaks in Apoptosis

Although DNA cleavage occurs in both self-driven and phagocytic phases, its mechanisms and resulting products are very different. Whereas a variety of executioner (cell-autonomous) nucleases participate in the initial phase of apoptotic cell disassembly, a single nuclease DNase II plays a fundamental role in the phagocytic phase of apoptosis, when cell corpses are removed by professional phagocytes – macrophages, or neighboring cells [1,8]. DNase II is present in lysosomes of phagocytizing cells and is essential for the final degradation of the engulfed DNA [1,2]. Its vital importance is evidenced by the fact that the enzyme is present in all animal cells and is highly conserved with close homologs of mammalian DNase II present in the invertebrates Caenorhabditis elegans and Drosophila melanogaster [9]. DNA cleavage by this acid deoxyribonuclease produces specific double-strand DNA breaks of 3’PO_4_/5’OH configuration, referred to as DNase II-type breaks [10,11]. Their end-group pattern is inverted, as compared to the 3’OH/5’ PO_4_ DNA breaks produced in the internal apoptotic phase by cell-autonomous executioner nucleases [1] and referred to as DNase I-type breaks [10,11]. The distribution of phosphate (PO_4_) or hydroxyl (OH) functional groups at the cleaved DNA ends provides important information about the enzyme which did the cutting and can be used in labeling its activity in tissue sections. For example, the enzymatic assays for labeling apoptotic breaks *in situ* focus exclusively on DNase I-type cleavage [12,13,14,15]. This configuration (3’OH/5’PO_4_ at the ends of the cut DNA fragments) is produced by the predominant apoptotic executioner nuclease CAD and other nucleases active in the self-autonomous phase of apoptosis [8,16,17]. As a result, the assays detect either 3’OH or 5’ phosphorylated DNA breaks and do not label DNase II-type breaks with terminal 5’OH. Therefore, in all of these assays, the cells with DNase II-type cleavage go undetected [11]. We decided to overcome this limitation and introduce the technique to selectively label DNase II-type cleavage, which is an obligatory type of cleavage produced in phagocytosed DNA. 

### 1.2. Sites of Generation of DNase II Breaks in Phagocytosis of Apoptotic Cells

**Figure 1:**
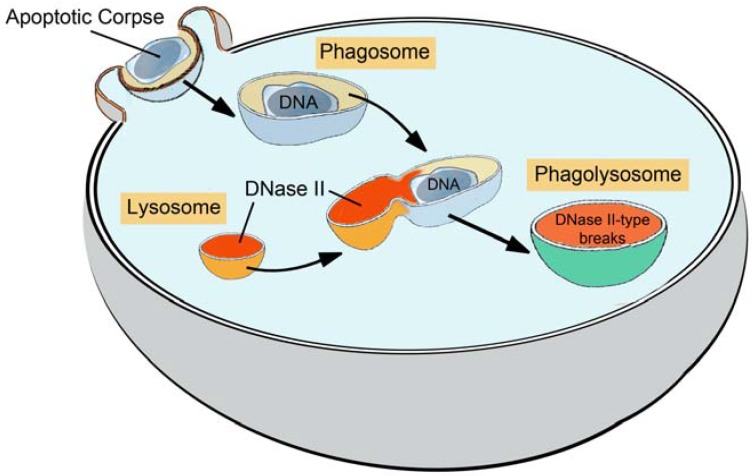
Lysosomes, phagosomes and phagolysosomes in clearance of apoptotic corpses.

After its synthesis in the endoplasmic reticulum, the DNase II enzyme is transported to the Golgi apparatus and then transfers and accumulates in the late endosomes, which mature into lysosomes [9,18,19]. These primary lysosomes contain no DNA substrate for DNase II to digest, so its activity cannot be visualized at this stage. The DNA substrate for digestion appears after the initiation of phagocytosis and is delivered by a phagosome, a separate vacuole formed around an engulfed apoptotic corpse (Figure 1). The phagosome is formed by the fusion of cellular membrane around the engulfed material, such as an apoptotic cell nucleus, etc. Compared to the primary lysosome, it is a larger, sack-like cellular compartment. At the start, it does not contain the digestive enzymes necessary for the lysis of the absorbed material. Later the phagosome fuses with primary lysosomes and acquires abundant hydrolytic enzymes. This leads to its transformation into a phagolysosome, which is the final site where the engulfed material is destroyed [18,19,20]. Phagolysosomes vary in size and shape, which is determined by the size and shape of the engulfed material (Figure 1). DNase II is solely responsible for DNA cleavage in the acidic conditions of a phagolysosome [21,22] and during this process it produces highly characteristic cuts which can serve as a marker of its activity. 

Here we present fluorescent probes which label this residual and stable molecular fingerprint of DNase II. The probes detect blunt-ended 5’OH DNA breaks specific for this acid deoxyribonuclease. The new probes make possible rapid and selective detection of the phagocytic phase of apoptosis in paraffin-embedded tissue sections.

### 1.3. Fluorescent Probes for Detection of DNase II Breaks *In Situ*

**Figure 2:**
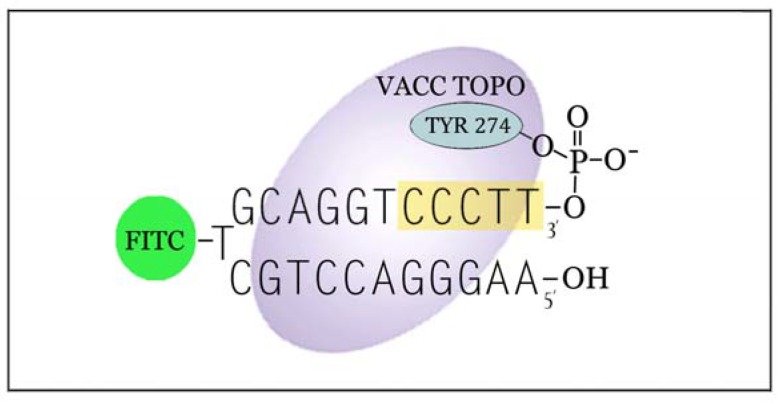
Fluorescent probe for detection of phagocytic phase of apoptosis: enzyme-substrate complex of vaccinia topoisomerase I and DNA. The probe consists of 3 parts: a protein (VACC TOPO), a hairpin-shaped oligonucleotide and a fluorophore. The protein and DNA parts of the probe are held together by a covalent phosphotyrosine bond that links Tyr274 of VACC TOPO to the terminal 3’T of the oligonucleotide. VACC TOPO bound to the CCCTT motif will religate the oligonucleotide to a double-strand DNA break possessing a complementary 5’OH blunt-end. Therefore the breaks of DNase II-type are detected specifically and directly.

From the enzymological perspective, our fluorescent probes are covalent intermediates formed between the enzyme vaccinia topoisomerase I (VACC TOPO) and fluorescently-labeled DNA (Figure 2). VACC TOPO is a type IB topoisomerase from vaccinia virus [23]. This 314-amino acid protein is one of the smallest topoisomerases known. It is capable of repeated cycles of cleavage-resealing of the phosphodiester DNA backbone. VACC TOPO can join two DNA molecules employing a mechanism different from those of ligases. In the reaction, the enzyme at first binds to double-stranded DNA having the CCCTT3’ recognition sequence and during the cleavage phase makes a cut at the 3’ end of the sequence, subsequently linking itself to the 3’ end of DNA. This creates a covalent enzyme-DNA junction, which is a transient step in its standard reaction cycle [24,25]. In normal conditions the enzyme then re-seals the break by re-ligating the strand back to the original DNA end with 5’OH, and thus restoring the original duplex DNA. However in our application, the enzyme-substrate complexes are prevented from re-ligation by removal of the original 5’OH acceptor DNA. Instead they are used as active and specific fluorescent probes for detection of 5’OH DNase II-type breaks. 

### 1.4. Approaches for Preparation of Reactive VACC TOPO Probes

**Figure 3:**
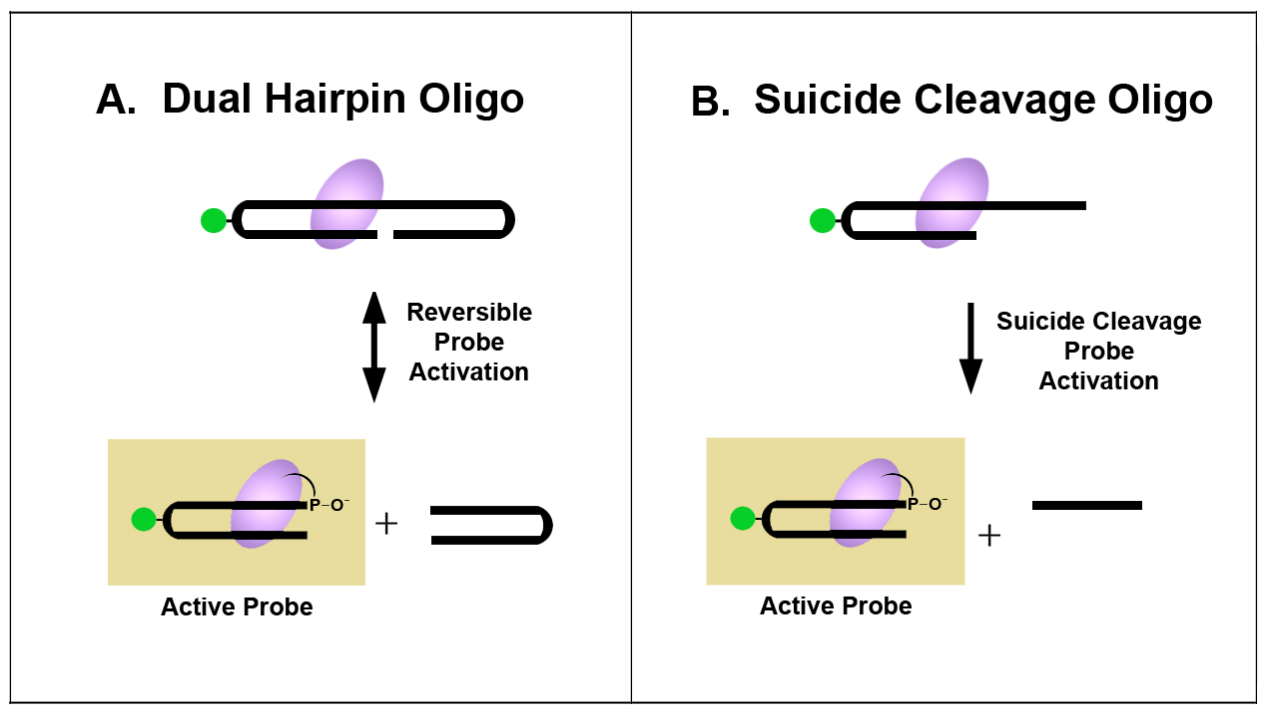
Approaches for preparation of fluorescent DNA-(3-phosphotyrosyl)-topoisomerase intermediates used as probes for detection of phagocytic phase of apoptosis. **A**. The dual hairpin “starting” oligo; **B**. Suicide cleavage approach; “starting” oligo with 12-base long 3’ tail.

All DNA ligases connect DNA strands carrying 3’OH groups to strands with 5’PO_4_ [14,26]. In this regard, VACC TOPO possesses a unique property being able to ligate to 5’OH DNA ends. No ligase can perform such a reaction. However, the ligation initiated by VACC TOPO is even more unlike the reactions performed by DNA and RNA ligases because it requires the enzyme to be specifically and covalently linked to the 3’ end of DNA prior to the ligation reaction. Therefore to form an active ligatable probe, the enzyme should initially react with a “starting” duplex DNA, then cleave it and attach to its 3’ end. All of our probes are intermediates of this reaction between VACC TOPO and fluorescent oligonucleotides, and can be prepared from different “starting” oligos (Figure 3). 

Our prototypical probes for labeling 5’OH double-strand DNA breaks were an offshoot of our work in bio-nanotechnology developing an oscillating nano-size device, which used VACC TOPO as a motor for driving the separation-religation of a dual labeled DNA part [27]. The construct exemplified a bio-enabled approach to the design of molecular devices and machines, and illustrated our notion that nano-size constructs that use mechanisms developed in the evolution of biological molecules are simpler and uniquely suitable for nanoscale environments. However when using the double-hairpin oligo as a starting material for the probe preparation, a significant part of the enzyme activity was wasted, as VACC TOPO constantly re-ligated and then re-cleaved the oligonucleotide (Figure 3A).

For that reason we re-designed the starting oligo and used a suicide cleavage approach which precluded re-ligation (Figure 3B). This permitted us to significantly simplify the assay and make it more cost-effective. For this we substituted the previously used oscillating double-hairpin with a much shorter “starting” oligo of a different configuration.

In the suicide cleavage oligonucleotide, the VACC TOPO recognition sequence CCCTT3’ is located in a hairpin with a 12-base long overhang on its 3’ end. The enzyme recognition site is positioned at the end of the duplex-forming part of the probe and on the edge of an unhybridized 12-base overhang (Figure 3). When VACC TOPO attaches to the probe, it cleaves the strand just after the recognition sequence. This cuts off the 12-base fragment which then permanently separates, leaving VACC TOPO attached to the 3’ end of the blunt-ended hairpin [11]. Now the oligonucleotide has a topoisomerase molecule strongly attached to its 3’ end. It can label DNase II-type breaks because VACC TOPO, which remains bound to the CCCTT motif, will religate the oligonucleotide to a double-strand DNA break possessing a complementary 5’OH blunt-end. When applied to tissue sections this probe specifically detects DNase II-type breaks. The labeling reaction proceeds in one direction in spite of the potentially reversible nature of the topoisomerase-based ligation (Figure 3A). This is because immediately after the probe ligates to a DNA break the topoisomerase reversibly separates from it [23]. Its reattachment to the same probe is prevented by the excess concentration of “starting” oligos, which preferentially react with the freed enzyme molecule. This creates a new active enzyme-substrate probe and leads to another round of labeling. The 12-base overhang on the probe is required because the enzyme will not cut a shorter strand [28] and will therefore be unable to attach to the probe and activate its 3’end. Here we present tests and applications of VACC TOPO probes in several tissue section models of the phagocytic phase of apoptosis employing normal and malignant immune system cells.

## 2. Results and Discussion

**Figure 4:**
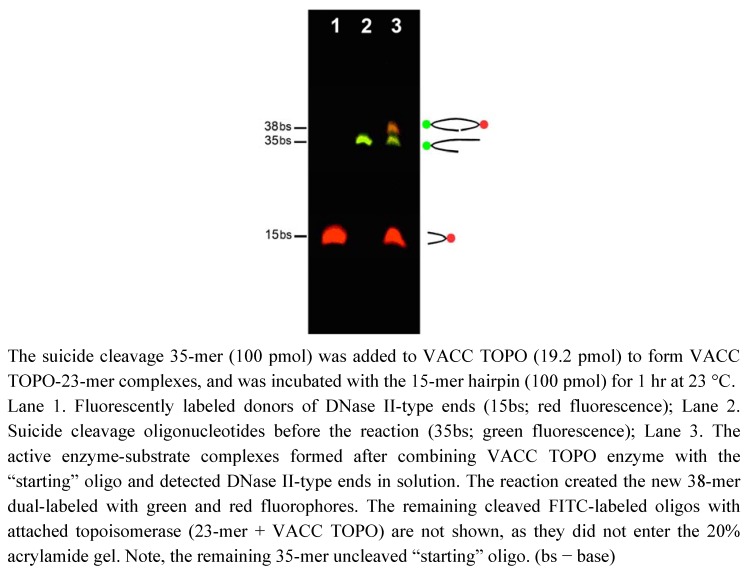
Detection of blunt-ended 5’OH DNA ends in solution by VACC TOPO probes produced by suicide cleavage (20% acrylamide gel; 165 V – 2.5 hrs).

Figure 4 shows generation of enzyme-substrate complexes by suicide cleavage and their specific reaction with DNase II-type ends in solution.

In these experiments, DNase II-type ends were provided by 15-mer hairpins (T_m_ = 46.7 °C), rhodamine-labeled at the apexes opposite to the open ends (*15bs band on lane 1*), and the 35-mers were used as suicide cleavage oligonucleotides (*35bs band on lane 2*). The activated probe was prepared by adding VACC TOPO enzyme to the FITC-labeled 35-mer “starting” oligonucleotides (23-base hairpin with 12-base 3’ overhang) (see Figure 3B). VACC TOPO cleaved the 12-base overhang and attached itself to the remaining 23-mer hairpin. It then ligated this FITC-labeled 23-mer to the rhodamine-labeled 15-mer hairpin producing a 38-mer oligo dual-labeled with FITC and rhodamine (*38bs band on lane 3*). 

The new probes were evaluated by using dexamethasone-treated apoptotic rat thymus. This apoptosis model was chosen because of the simultaneous presence of both DNase I- and DNase II-types of breaks [27]. In addition, apoptosis and phagocytosis in thymus occur in the absence of inflammation and involve two well-defined types of cells: thymocytes and macrophages [16,27]. The glucocorticoid treatment used in this model leads to massive cell death of cortical lymphocytes within a 24-hour period, whereas the core areas containing different types of cells remain unaffected. The apoptotic corpses in thymus are subsequently cleared by cortical macrophages. 

**Figure 5:**
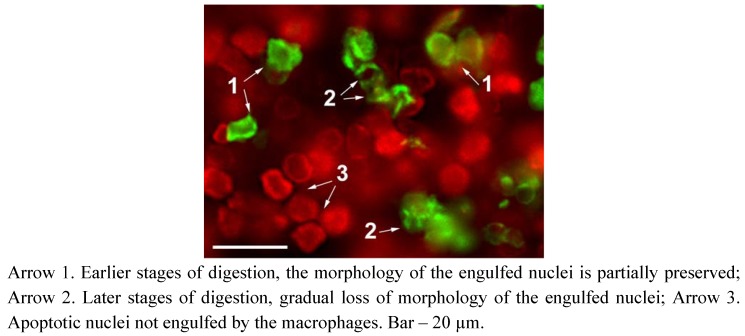
VACC TOPO labels phagolysosomes in cortical macrophages digesting nuclei of apoptotic thymocytes. Green fluorescence – VACC TOPO probe labeling of DNase II-generated breaks in the phagocytic stage of apoptosis. Red fluorescence – ISL probe labeling of DNase I-type breaks produced by CAD endonuclease in the self-autonomous phase of apoptosis [17].

**Figure 6:**
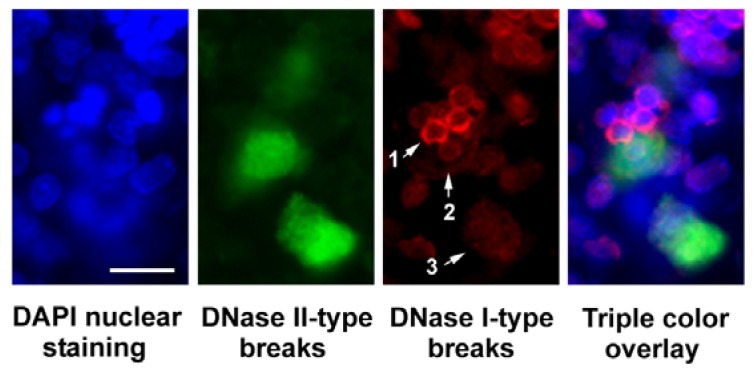
In the engulfed apoptotic nuclei the DNase II-type breaks gradually substitute the initial DNase I-type cleavage produced at the earlier, self-autonomous phase of apoptosis. Bar – 20 µm.

**Figure 7:**
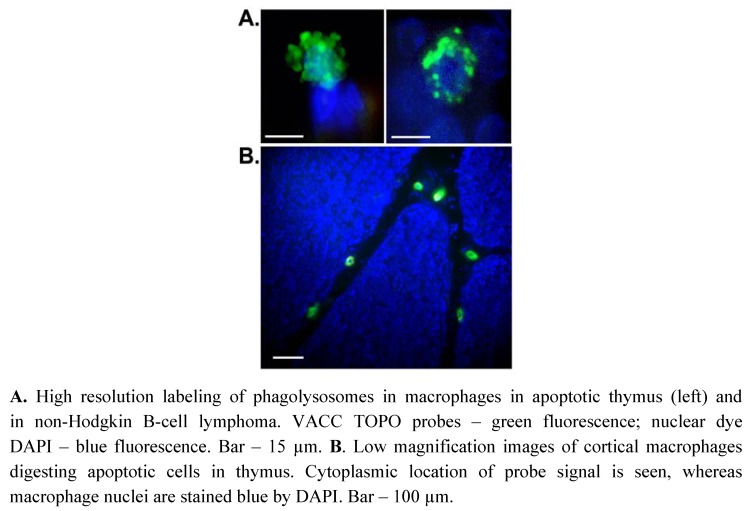
VACC TOPO probes in high (**A**) and low (**B**) magnification applications.

The sections of apoptotic thymus were dual- and triple- stained by using the VACC TOPO probes; and also by *in situ* ligation (ISL) [14,15]; and by the nuclear stain DAPI (4’,6-diamidino-2-phenylindole) (blue). The results of these experiments are illustrated in Figure 5, Figure 6 and Figure 7.

Figure 5 presents a high magnification image of the apoptotic cortical zone of thymus, dual-labeled by VACC TOPO probes for DNase II-type breaks (green fluorescence) and by *in situ* ligation for DNase I-type breaks (red fluorescence).

The figure shows multiple nuclei of apoptotic thymocytes with extensive DNase I-type cleavage (red fluorescence). The nuclei display characteristic apoptotic morphology with doughnut-shaped patterns of nuclear chromatin condensation. These morphological changes of apoptotic nuclei (chromatin condensation and margination) were induced during the cell-autonomous phase before phagocytosis. The VACC TOPO probe instead marked areas of DNase II activity (green fluorescence), and revealed apoptotic cell nuclei engulfed by macrophages. The engulfed nuclear material is seen in various stages of digestion in the phagolysosomes of cortical macrophages.

VACC TOPO probes selectively label phagolysosomes because these are the only structures within cells where the DNase II reaction is carried out. Although the DNase II enzyme can also be found in primary lysosomes, they do not contain DNA and cannot be imaged by our probes. Besides, DNase II is inactive at the normal intracellular pH 7.4, which is maintained outside of the acidic environment of lysosomes and phagolysosomes. Its pH activity range is 4.0-6.5, with the optimum at pH 4.8 [22]. The enzyme is strongly pH sensitive and looses 85% of activity at pH 6.5, with complete inactivation at higher pH [29]. So in normal conditions, its reactions are sequestered and occur in phagolysosomes where it is used to digest the engulfed DNA.

The VACC TOPO probes cannot detect the DNase II enzyme *per se* in the absence of digested DNA, therefore the only organelle which the probe can label, in the apoptosis models we use, is a phagolysosome containing both the active enzyme and its substrate. 

Figure 5 (arrow 1) shows the engulfed nuclei with the preserved “doughnut-shape” morphology, which indicates the initial stages of digestion, shortly after the phagolysosome formation and the DNase II delivery. Nevertheless, the predominant DNA cleavage at this stage is already of DNase II-type (green fluorescence). Compared to the DNase I generated signal, the DNase II signal is significantly stronger reflecting the higher extent of DNA degradation in phagolysosomes. Although the general shape of the nuclei is still maintained, they appear distorted and flattened with invaginations and breaks in nuclear envelopes. These probably occur due to the simultaneous action of lysosomal proteases and lipases on the nucleolemma.

The later stages of digestion of phagocytosed apoptotic nuclei [Figure 5 (arrow 2)] show complete breakage of the nuclear membrane and dissipation of the apoptotic nuclei into separate pieces. The multitude of the smaller nuclear bits becomes observable, all displaying strong DNase II signal, which in some areas becomes diffuse indicating the complete dissolution of the structures. 

It was conclusively demonstrated that DNase II is a single source of DNA cleavage in engulfed and digested DNA and no other deoxyribonuclease is active during the phagocytic clearance [22]. The measurements, performed in cell extracts prepared from *DNase II^-/-^* tissues and assessed under acidic conditions of lysosomal digestion, showed complete absence of any DNase activity, confirming that DNase II is the only acid DNase present in lysosomes and is solely responsible for the extensive DNA cleavage of engulfed apoptotic DNA [22].

Although no other breaks but DNase II-type (3’PO_4_/5’OH) are produced in phagolysosomal digestion, the cells often have abundant DNA breaks of DNase I-type (3’OH/5’PO_4_) at the start of phagocytosis (Figure 5). These are generated in the preceding, self-autonomous phase of apoptosis by the apoptotic nucleases, such as caspase-activated deoxyribonuclease (CAD) [1]. The numbers of these breaks increase in the self-autonomous phase from about 50,000 per genome at the initial high-molecular-weight DNA degradation to 3 × 10^6^ during later internucleosomal DNA fragmentation stage [30].

The fate of these DNase I-type breaks in the phagocytic phase in mammalian cells was not investigated before. In the meantime, this type of DNA damage is almost exclusively used by the enzymatic *in situ* labeling techniques, such as TUNEL or ISL, as a marker of apoptotic cells [12,13,14,15]. Therefore it would be important to examine the stability of this marker and its persistence in the phagocytic phase until complete disintegration of apoptotic cells.

In line with this, we used the model of dexamethasone-treated thymus to investigate what happens to the pre-existing DNase I-type breaks in the waste-management phase, after apoptotic nuclei are phagocytosed.

The thymic model is particularly suitable for this study because it permits observing different stages of apoptotic progression and clearance in the same section. In experiments the thymus sections were double-stained for DNase I- and DNase II-type DNA breaks by using ISL and VACC TOPO probes. All cellular nuclei were imaged by fluorescent blue dye DAPI. The results of these experiments are illustrated in Figure 6.

Figure 6 demonstrates that the DNase I-type breaks, produced during self-autonomous DNA fragmentation, persist through the initial and even intermediate stages of phagocytosis, being gradually substituted by DNase II-type cleavage. The initial contact of a macrophage with a group of apoptotic nuclei is marked by *arrow 1* in Figure 6, and presents the earliest stage, before the formation of a phagolysosome. At this stage the breaks in the nuclei are exclusively of DNase I-type (complete absence of green fluorescence). They also maintain the doughnut pattern of intranuclear distribution, characteristic for the self-autonomous apoptotic phase. The later stage of digestion is exemplified by the engulfed nucleus deep inside the macrophage, which is marked by *arrow 2* in Figure 6. The nucleus is located in the zone with extensive generation of DNase II breaks (green fluorescence), however the initial DNase I-type signal showing the same pattern can still be seen, although weakened (red fluorescence *arrow 2*). This gradual disappearance of DNase I-type signal likely occurs due to the creation of the new DNase II-type breaks close to the ends of the apoptotic DNA fragments, which results in the destruction of the earlier DNase I-type configuration. At the next stage (*arrow 3* in Figure 6) the faint signal indicating DNase I-type breaks can be observed in the fragmented remains of nuclear material inside phagocytes, before they finally disappear being overlaid by extensive DNase II-type cleavage (green fluorescence).

Thus, if apoptotic nuclei underwent extensive DNA fragmentation in the self-autonomous phase, they can still be labeled after their engulfment and being inside the macrophages by using techniques which detect DNase I-type cleavage, such as *in situ* ligation or TUNEL. However, this residual signal is a relic of the earlier stage and eventually disappears during digestion. The actual activity of phagocytic DNA cleavage cannot be assessed by this marker. Reliance on such labeling alone makes it difficult, if not impossible, to distinguish phagocytosed apoptotic cells from non-phagocytosed. However, as we demonstrate in Figure 6, such distinction can be accomplished by using the VACC TOPO probes.

In sum, VACC TOPO probes are advantageous for studies of apoptosis because they can label a phagocytic marker, such as DNase II-type breaks bearing 5’OH, undetectable by the other enzymatic *in situ* techniques − TUNEL or *in situ* ligation. However their utility can be increased by combining them with either TUNEL or ISL, and thus providing a complete characterization of DNA ends *in situ*. Such characteristics include labeling of 3’OH ends by TUNEL [12], 5’PO_4_ ends by ISL [14,15] and now detection of 5’OH ends by VACC TOPO. 

VACC TOPO probes provide high resolution images and can be broadly applied to image phagolysosomes during apoptotic cell clearance, not only in normal thymus but in the other immune tissues in pathological situations, such as in malignant lymphomas (Figure 7A).

It is also important that the fluorescence signal from VACC TOPO probes is strong and sufficient for low magnification observations and counting of phagocytizing cells (Figure 7B). Such counts provide a quantitative measure of the phagocytic activity in different tissue samples helpful in evaluation and comparison of pathology specimens.

In line with this, we applied the probes to the detection and counting of phagocytic cells in a tissue section array of human lymphomas. The goals were to test the probes in health-relevant models of apoptotic cell engulfment and to assess the convenience of the new probes’ usage for visualization and assessment of vastly different levels of phagocytic cell activity. Malignant lymphomas were chosen because various malignant lymphoid tissues differ considerably in the extent of phagocytosis of their apoptotic cells by macrophages. These differences are well-documented and range from high to extremely low [31,32,33]. In experiments we used lymphoma tissues with different rates of phagocytosis obtained from BioChain as an array of the uniformly cut 1.1 mm circular sections of 4 µm thickness. The sections formed a test panel which permitted using cellular morphology for supplementary verification of phagocytizing cells. 

The high grade mucosa-associated lymphoid tissue (MALT) lymphoma was employed as a source of the most intense phagocytic activity because this tumor, derived from a background of inflammatory disease, is characterized by massive recruitment of phagocytes [34,35] and the mantle cell lymphoma was used as a tumor with extremely low incidence of phagocytic clearance [31]. The group also included the Non-Hodgkin B cell lymphoma and Hodgkin lymphoma as examples of intermediate to high phagocytic activity [31,32,33].

**Figure 8:**
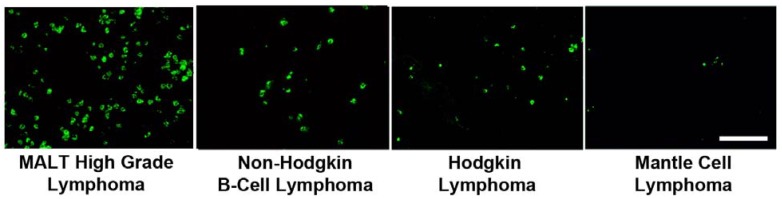
Various intensity of phagocytic cell clearance visualized by VACC TOPO in sections of human lymphomas. Bar – 100 µm

When applied to lymphoma sections the probes revealed vastly different incidence of phagocytic clearance in lymphomas (Figure 8). As expected the activity was the highest in MALT lymphoma and the lowest in the mantle cell lymphoma with intermediate levels displayed by the B-cell and Hodgkin lymphomas. The phagocytizing cells in all tissues were brightly fluorescent and could be counted throughout without difficulties.

The complete counts of phagocytizing cells in the each of the 1.1 mm circular sections were obtained. They were: MALT lymphoma: 798; Non-Hodgkin B Cell lymphoma: 108; Hodgkin lymphoma: 55; Mantle lymphoma: 3. The results are in agreement with the previously published assessments of phagocytic activity in these tumors [31,32,33,34,35]. The differences in phagocytic clearance in these lymphomas could be explained by the various rates of apoptosis in these tumors [36,37]. However, the impairment of phagocytic function resulting in its suppression in some tumors and activation the others is also a prospective contributing factor [31,32,33]. The exact mechanisms of these processes are under study at this time.

The ease of detection and the clarity of the signal provided by the new probes demonstrate their usefulness in tissue analysis of phagocytic engulfment. Their advantage is highlighted by the fact that antibody-based macrophage or other phagocytizing cell markers would not be able to provide similar result in the lymphoma models. This is because the infiltration rates of macrophages in lymphomas do not correlate with phagocytic activity. The mantle lymphoma, for example, has very high levels of macrophage infiltration, while having the lowest levels of phagocytic clearance [31]. 

In many tumors the rate of macrophage infiltration depends more on the grade of malignancy and on the proliferation rate of the tumors and does not indicate more intense phagocytosis [31,32,33]. This indicates that antibody-based immunohistochemical markers of macrophages, if used alone, cannot provide a reliable picture of phagocytic reactions in lymphomas. The new fluorescent VACC TOPO probes which selectively label phagocytizing cells would be very useful in such types of tissue analyses.

## 3. Experimental

### 3.1. Materials and Instruments

12-base tailed “starting” oligonucleotide labeled with a single fluorescein was synthesized and PAGE purified by Integrated DNA Technologies, Inc. On receipt it was diluted with bidistilled water to 1.13 µg/µL (100 pmol/µL) stock concentration and stored at −20 °C protected from light. The oligonucleotide sequence was:


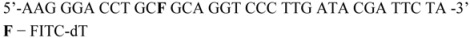


Proteinase K was from Roche Molecular Biochemicals and was used as 20 mg/mL stock solution in distilled water; Vaccinia DNA topoisomerase l − 3000 U/μL was obtained from Vivid Technologies, Inc.; Vectashield with DAPI was obtained from Vector Laboratories; Lymphoma tissue sections were purchased from BioChain Institute; 5 μm-thick rat thymus sections were cut from paraformaldehyde-fixed, paraffin-embedded tissue blocks of a rat thymus from dexamethasone-treated rat [16]. Sodium bicarbonate buffer contained 50 m*M* NaHCO_3_, 15 m*M* NaCl, pH 8.2.

Fluorescence Olympus IX-70 microscope with Chroma Technology band-pass filter set was used: FITC excitation D490/40, emission 520/10; DAPI excitation D360/40, emission 460/20. Images were recorded by an Olympus EVOLT digital SLR and a MicroMax digital video camera system (Princeton Instruments, Inc.)

### 3.2. Preparation of Fluorescent Enzyme-Substrate Complexes and Labeling of Phagocytic Cells in Tissue Sections

To prepare the sections for the labeling reaction, they were dewaxed in xylene for 15 min, and passed through graded ethanol concentrations: 96% Ethanol – 2 × 5 min; 80% Ethanol – 5 min; water – 2 × 5 min. The sections were then treated with Proteinase K for 10 min at room temperature (23 °C) in a humidified chamber. 100 μL of a 50 μg/mL solution was used per section. Subsequently the sections were rinsed in distilled water for 2 × 10 min.

To prepare fluorescent enzyme-substrate complexes for the labeling reaction, while sections were rinsed in water, 100 pmoles of the tailed “starting” oligonucleotide were combined in a vial with 20 pmoles (0.66 µg) of vaccinia topoisomerase I in solution of 50 mM Tris-HCL, pH 7.4. The solution was incubated at room temperature for 15 min to allow for probe activation and was then used in 25 µL aliquots per section. In the case of arrays, a larger volume was used for the entire section to be covered.

The sections were covered with plastic coverslips and incubated for 18 hours at room temperature (23 °C) in a humidified chamber protected from light. The sections were gently immersed vertically in a Coplin jar containing water at room temperature to remove the coverslips and were then washed 3 × 10 min in distilled water and rinsed with sodium bicarbonate buffer. They were subsequently covered with Vectashield with DAPI antifading solution, coverslipped and analyzed under a fluorescence microscope. Phagocytizing cells displayed cytoplasmic green fluorescence, confined to phagosomes and blue-fluorescing nuclei.

For the dual staining using *in situ* ligation and VACC TOPO probes, the sections were initially incubated with VACC TOPO probes and after incubation were co-labeled by *in situ* ligation [14]. The VACC TOPO solution was aspirated sections were rinsed in water before applying the reaction mix (25 µL) containing 66 mM Tris-HCl, pH 7.5, 5 mM MgCl_2_, 0.1 mM dithioerythritol, 1 mM ATP, 15% polyethylene glycol-8000, ISL probe (blunt-ended hairpin) (35 mg/mL) and T4 DNA ligase (250 U/mL). Sections were incubated in a humidified box (16 h, 23 °C). They were then briefly washed in water. Sections were then counterstained with 4,6-diamidino-2-phenylindole (DAPI)

The ISL probe labeled with a single rhodamine was synthesized and PAGE purified by Integrated DNA Technologies, Inc. The probe sequence was: 5’-GCG CTA GAC C**R**G GTC TAG CGC-3’; **R** = tetramethylrhodamine-dT

## 4. Conclusion

Here we describe a new and improved fluorescent probe for apoptosis research. The probe visualizes the phagocytic (waste-management) phase of apoptotic DNA degradation. It labels blunt-ended 5’OH DNA breaks in tissue sections and marks phagolysosomes digesting nuclear material of the engulfed apoptotic cells. This enables investigation of cell death as a broad reaction continuing beyond the individual cell program and requiring participation of other cells. 
